# Reducing social disparities in child emotional and behavioral problems by hypothetical physical activity and screen time interventions

**DOI:** 10.1007/s00127-025-03036-6

**Published:** 2026-01-19

**Authors:** María Rodriguez-Ayllon, Pauline W. Jansen, Jeremy A. Labrecque, Clair A. Enthoven

**Affiliations:** 1 Department of Child and Adolescent Psychiatry/Psychology, Erasmus MC University Medical Center, Rotterdam, 3000 CA The Netherlands; 2 Biomedical Research Institute of Malaga (IBIMA Platform Bionand), Malaga, Spain; 3 Prevention and Health Promotion Research Network (redIAPP) & Chronicity, Primary Care and Health Promotion Research Network, (RICAPPS), ISCIII, Madrid, Spain; 4 Department of Psychology, Education and Child Studies, Erasmus School of Social and Behavioural Sciences, Erasmus University, Rotterdam, The Netherlands; 5 The Generation R Study Group, Erasmus University Medical Center, Rotterdam, The Netherlands; 6 Department of Epidemiology, Erasmus University Medical Center, Rotterdam, The Netherlands

**Keywords:** Exercise, Sports, Mental health, Social disparities, Youth, Childhood

## Abstract

**Purpose:**

To estimate how social disparities in child psychiatric symptoms might change following hypothetical interventions targeting sports, outdoor play, and screen time at age 10.

**Methods:**

We used data from 9,778 children of the Generation R Study, a prospective population-based cohort in Rotterdam, the Netherlands. Social inequality variables included sex, maternal education, and migration background. Primary caregivers filled out the validated Child Behavior Checklist to report on children’s internalizing and externalizing symptoms at the age of 13. The hypothetical interventions (i.e., outdoor play, sports participation, and screen time) were parent-reported at age 10. We used sequential G-estimation to estimate the inequality with and without the hypothetical intervention.

**Results:**

Children with migration backgrounds (46.3%) and low maternal education (53.3%) were associated with relatively more internalizing and externalizing symptoms than peers, with disparities of 0.125 and 0.177 standard deviations, respectively. Girls had more internalizing symptoms (0.106 SD), while boys had more externalizing symptoms (0.154 SD). Increasing sports participation reduced disparities in internalizing symptoms linked to maternal education (β = -0.014; 95% CI: -0.024, -0.003), while outdoor play and screen time interventions showed limited effects. None of the hypothetical interventions led to a statistically significant reduction in social disparities in externalizing symptoms.

**Conclusions:**

This study underscores the persistence of sex, cultural, and socioeconomic disparities in youth mental health. While sports participation showed a potential effect in reducing disparities in internalizing symptoms, its impact on externalizing symptoms and other interventions was negligible. Future efforts should focus on identifying more effective strategies for addressing these disparities.

**Supplementary Information:**

The online version contains supplementary material available at 10.1007/s00127-025-03036-6.

## Introduction

Globally, mental disorders account for 15% of the disease burden among adolescents aged 10 to 19, meaning that one in seven individuals in this age group is affected [1]. These conditions can have enduring negative consequences, such as school dropout and poor physical health. Notably, the age of onset of most mental disorders, including depression and anxiety, is before mid-adolescence [2]. This highlights the urgent need for early preventive interventions to safeguard young people’s mental health and address potential issues before they emerge.

Studies consistently demonstrate that social disparities, particularly those linked to socioeconomic status (SES), heighten the risk of psychiatric symptoms in childhood [3]. Key SES indicators, such as parental education and migration background, are closely tied to these disparities. For instance, children from families with less-educated parents or from migrant families face higher rates of mental health challenges, underscoring the profound impact of SES on psychological development [4, 5]. In addition to these social factors, sex, defined as sex assigned at birth, is another critical risk factor for psychiatric symptoms. Research indicates that girls are more prone to internalizing issues, such as depression and anxiety [6], while boys are more likely to exhibit externalizing behaviors, such as conduct problems or Attention-Deficit/Hyperactivity Disorder (ADHD) [7]. Although our study focused on sex as a measure, it is important to acknowledge that gender, which refers to the social and cultural roles people identify with, may have a different association with psychiatric symptoms. Overall, addressing these disparities is essential for improving mental health outcomes among young people from low-education and migrant backgrounds, as well as those affected by sex differences. While factors like stress or limited social support are well-established contributors, further research is needed to identify specific, actionable protective factors in children and adolescents. Such insights will enable the development of targeted interventions to reduce social disparities in youth mental health effectively.

Engaging in physical activities, such as sports, serves as a powerful protective factor against the development of psychiatric symptoms through multiple pathways. On a molecular level, it helps reduce cortisol levels; in the brain, it enhances gray matter volume in critical regions such as the amygdala and hippocampus; and psychosocially, it enhances social skills and self-esteem which may alleviate symptoms of depression and anxiety [8, 9]. Although less consistently shown, evidence also suggests that spending less time on screens is associated with better mental health during childhood and adolescence [10]. Despite these well-documented benefits, one in three children and adolescents fails to meet the physical activity recommendations of the World Health Organization (WHO) [11]. This problem is even more pronounced among children from families with lower education levels or migration backgrounds, who are less likely to participate in sports [11] and are more prone to excessive recreational screen time compared to peers from higher socioeconomic backgrounds. Furthermore, patterns of physical activity and screen use differ by sex: boys tend to be more physically active but also spend more time on screens [11, 12], whereas girls are generally less active [11]. While previous research has highlighted these sociodemographic disparities, it remains unclear whether differences in physical activity and screen time help explain socioeconomic and sex-related disparities in mental health. To address this gap, the present study aimed to estimate how social disparities in child psychiatric symptoms (i.e., internalizing and externalizing symptoms) at age 13 might change following hypothetical interventions targeting physical activity and screen time at age 10, using a sequential G-estimation model—a question with direct implications for selective preventive interventions.

## Methods

### Study design and population

We used data from the Generation R Study, a prospective population-based birth cohort conducted in Rotterdam, the Netherlands. Almost ten thousand pregnant women living in Rotterdam, the Netherlands were enrolled in the study. Their estimated delivery date was between April 2002 and January 2006, and they gave birth to 9,898 children who have been followed since. A detailed description of the study design is provided elsewhere [13, 14]. The Medical Ethics Committee of Erasmus Medical Centre approved all study procedures.

The current study used data from two time points, when children were around 10 and 13 years old. Data of all *N* = 9,898 children were included in the primary analyses. The participants were on average 13.6 (SD = 0.4) years old when internalizing and externalizing symptoms were measured. Around half of the participants were male (50.7%), almost half of them had a migration background (46.3%), and more than half had mothers with vocational training or less (53.3%). The full general characteristics of the cohort are shown in Table 1. The social disparities (i.e., stratified by sex, migration background, and maternal education) in outdoor play, sports participation, screen time, and internalizing and externalizing symptoms are shown in Tables S1, S2, and S3, respectively.

**Table 1:** Characteristics of the cohort

	Overall (*N* = 9,898)	Sample for sensitivity analyses (*N* = 3,413)
**Child sex**		
Boy	4,937 (50.7%)	1710 (50.1%)
Girl	4,808 (49.3%)	1703 (49.9%)
N	9,745	3,413
**Migration background**		
No	4,948 (53.7%)	2405 (70.5%)
Yes	4,273 (46.3%)	1008 (29.5%)
N	9,221	3,413
**Maternal education**		
Lower	4,858 (53.3%)	1214 (35.6%)
Higher	4,264 (46.7%)	2199 (64.4%)
N	9,122	3,413
**Child age at psychiatric measurement**		
Mean (SD)	13.56 (0.40)	13.52 (0.35)
N	4,961	3,413
**Outdoor play (hrs/week)**		
Mean (SD)	5.63 (4.65)	5.57 (4.55)
N	4,811	3,413
**Sports participation (hrs/week)**		
Mean (SD)	2.60 (1.59)	2.67 (1.54)
N	4,905	3,413
**Screen time (hrs/week)**		
Mean (SD)	17.29 (11.91)	16.50 (10.82)
N	4,424	3,413
**Parent reported internalizing symptoms (sumscore)**		
Mean (SD)	5.63 (5.81)	5.39 (5.60)
N	4,721	3,413
**Parent reported externalizing symptoms (sumscore)**		
Mean (SD)	4.22 (5.28)	4.07 (5.10)
N	4,709	3,413
**Self-reported internalizing symptoms (sumscore)**		
Mean (SD)	8.78 (7.15)	8.53 (7.05)
N	4,516	3,253
**Self-reported externalizing symptoms (sumscore)**		
Mean (SD)	7.03 (5.33)	6.92 (5.25)
N	4,503	3,244

*Hrs * Hours, *N * Number of participants, *SD * Standard deviation

### Social disparities

For assessing social disparities, sex assigned at birth, migration background, and maternal education were used. Sex assigned at birth was obtained from medical records. Migration background and maternal education were assessed by questionnaire. The child was considered to have a migration background if one of their parents was born abroad [14]. Maternal education was defined as the highest completed education. Two categories were formed, indicating low (from no education to high school or vocational training) and high educational attainment (from higher vocational education to university).

### Outdoor play, sports participation, and screen time

Information on outdoor play, sports participation, and screen time was obtained from primary caregiver reports administered when children were 10 years old. The questionnaires for the primary caregiver were mostly completed by the mother (97%). To assess the level of physical activity, informants indicated both the frequency (number of days per week) and duration (minutes per day) of the child’s engagement in (i) sports participation, and (ii) outdoor play. Sports considered included soccer, hockey, basketball, handball, korfball, tennis, judo, karate, gymnastics, jazz ballet, etc. School sports activities, such as physical education lessons and swimming lessons, were not included in these physical activity variables. The hours per week spent on outdoor play and sports participation were calculated using the following formula: weekly time spent on the activity = (days per week) * (hours per day) [15].

Respondents were also asked to indicate the number of days and hours per day their child: (i) watches television (including videos/DVDs) and (ii) uses a computer or similar device (including video games). Screen time was assessed separately for weekdays and weekend days, and then combined to estimate the total hours per week spent on each activity. A total weekly screen time score was calculated by adding the hours of playing video games and watching television.

We decided on a hypothetical cutoff based on a combination of guidelines, categories available in our questionnaires, and the distribution of the data [16]. We set screen time, sports participation, and outdoor play to specific levels in each hypothetical intervention. Screen time was set to ≤ 2 h/day, outdoor play was set to ≥ 2 h/day, and sports participation was set to ≥ 2 h/week.

### Mental health

Primary caregivers filled out the validated Child Behavior Checklist (CBCL/6–18) around the child aged 10 and 13 years to report on children’s mental health problems [17, 18]. The CBCL consists of 112 items and evaluates emotional and behavioral symptoms in the preceding 6 months. Items are scored on a 3-point Likert scale (0 = not true, 1 = somewhat or sometimes true, and 2 = very true or often true). We examined the CBCL broadband subscales of internalizing problems (i.e., depression, anxiety, somatic symptoms) and externalizing symptoms (i.e., conduct problems, rule-breaking behavior, ADHD) by averaging the items related to those scores while allowing for 25% missing items. Cronbach’s alphas were 0.84 and 0.87 for internalizing problems and 0.87 and 0.88 for externalizing problems at respectively ages 10 and 13 years [19]. We additionally examined child self-reported internalizing and externalizing symptoms using the Brief Problem Monitor (BPM) at age 10, and the Youth Self Report (YSR) at age 13 years. The validated BPM is an abbreviated version of the YSR, consisting of 19 items, while the validated YSR contains 112 items [20, 21]. Cronbach’s alphas were 0.72 and 0.88 for internalizing symptoms, and 0.63 and 0.82 for externalizing symptoms at respectively ages 10 and 13 years, respectively [19].

### Potential confounders

At the age of 10, the child’s height and weight were measured at the research center, and body mass index (BMI) was calculated and standardized according to the Dutch reference growth curves (https://growthanalyser.org) [22]. At the age of 5, a non-verbal intelligence quotient (IQ) was assessed using the *Snijders-Oomen Niet-verbale intelligentie Test- Revisie (SON-R 2.5–7)* [23].

### Statistical analysis

All analyses were conducted in R statistical software version 4.2.1. Prior to analyses multiple imputations were performed to replace the missing values (ranging from 1.5% to 55.3%) using multivariate imputation by chained equations [24]. We created 30 imputed datasets with 100 iterations. Next to our variables of interest, the following variables were added as auxiliary variables to improve imputation because they have a low amount of missing data: household income, parental weight and height, paternal age at inclusion, gestational age at birth, birth weight, parity, and apgar score at 5 min [25].

Firstly, we determined the socioeconomic (education and migration background) and sex disparities in internalizing and externalizing problems using linear regression models, adjusted for age. Secondly, we constructed an ‘intervention’ model by estimating the effect of the hypothetical interventions (e.g., outdoor play) on the outcome (e.g., internalizing symptoms) using linear regression while adjusting for potential confounders that were selected using causal graphs and based on the relevant literature. Thirdly, the estimates of the ‘intervention’ model were used to estimate the change in mental health problems if all participants would have had the intervention using G-estimation [16, 19]. Finally, by comparing the inequality before and after the hypothetical interventions, the absolute change in social inequality was estimated, and the corresponding 95% confidence intervals (CIs) were calculated using bootstrapping with 1000 iterations.

Two sensitivity analyses were conducted. Firstly, we repeated the analyses using self-reported internalizing and externalizing symptoms instead of parent-reported outcomes. Secondly, we only included participants with complete data on the variables of interest, namely: sex, maternal education, migration background, internalizing and externalizing symptoms at the age of 13 years, and outdoor play, sports participation, and screen time at the age of 10 (*N* = 3,413).

The four steps of the statistical analyses rely on three causal assumptions: (I) Exchangeability, meaning that there is no residual confounding or selection bias between the proposed intervention and the measure of psychiatric symptoms. Therefore, we adjusted for child age at intervention measurement, child age at outcome measurement, IQ at age 5, standardized body mass index at age 9, and pre-existing psychiatric symptoms at age 9. We additionally adjusted for maternal education and migration background in the analyses with sex, for maternal education and sex in the analyses with migration background, and for sex and migration background in the analyses with maternal education. To avoid selection bias due to dropout, we included the whole sample (*N* = 9,898) and imputed all missing values (see Table 1 for the amount of missing data per variable); (II) Positivity, meaning that the probability of receiving any level of the intervention (i.e., outdoor time, sports, and screen time) conditional on the covariates must be greater than 0. We estimated the generalized propensity scores on the non-imputed dataset using the estimate_gps function with the non-parametric kernel approach of the CausalGPS R package [26]. The generalized propensity scores were all greater than 0; (III) Consistency assumes that the interventions are well-defined. This is unlikely to be the case for our lifestyle interventions, because screen time, for instance, does not take into account the content of the activity (e.g., gaming or schoolwork), nor does it specify whether a parent or peer is involved (e.g., gaming with a friend or receiving assistance from a parent during schoolwork). Consequently, the observed estimates will depend on how our participants define screen time, outdoor play, and sports participation.

## Results

### Physical activity and screen time levels

Boys had more outdoor play (6.09 h/week vs. 5.18 h/week), sports participation (2.84 h/week vs. 2.36 h/week) and screen time (18.5 h vs. 16.1 h/week) than girls. Children without a migration background had more outdoor play (5.78 h/week vs. 5.34 h/week), engaged more frequently in sports (2.82 h/week vs. 2.19 h/week) and had less screen time (15.4 h/week vs. 21.0 h/week) than those with a migration background. And children of highly educated mothers reported more outdoor play (6.14 h/week vs. 5.30 h/week), more sports participation (2.88 h/week vs. 2.17 h/week), and less screen time (14.7 h/week vs. 21.3 h/week) than those whose mothers had lower education levels.

### Internalizing symptoms

Girls had a 0.106 (95% CI: 0.066, 0.145) standard deviation more internalizing symptoms compared to boys. Similarly, children with a migration background had 0.125 (95% CI: 0.085, 0.165) standard deviation more internalizing symptoms than those without, and children with a lower educated mother had a 0.177 (95% CI: 0.138, 0.216) standard deviation more internalizing symptoms than those with a higher educated mother.

In the main analyses, hypothetically increasing outdoor play or reducing screen time did not significantly reduce the disparities in internalizing symptoms (Table 2). However, hypothetically increasing sports participation significantly reduced the disparities for maternal education (β = -0.014; 95% CI: -0.024, -0.003) (Table 2). In the sensitivity analyses, results were virtually the same in the complete case analyses (β = -0.017; 95% CI: -0.034, -0.000) and when using self-reported internalizing symptoms (β = -0.024; 95% CI: -0.036, -0.012). See Table S4 and Table S5, respectively. In contrast to the main analyses, the sensitivity analyses showed that hypothetically increasing sports participation also reduced the disparities in self-reported internalizing symptoms for sex (β = -0.016; 95% CI: -0.029, -0.003) and migration background (β = -0.012; 95% CI: -0.025, 0.000). Additionally, hypothetically reducing screen time reduced the disparities in self-reported internalizing symptoms for maternal education (β = 0.030; 95% CI: 0.009, 0.052; Table S5).

**Table 2:** Reductions in sex, migration background and maternal education disparities on internalizing and externalizing symptoms at the age of 13 from hypothetical outdoor play, sports participation and screen time interventions in children at age 10

Internalizing symptoms						
	Sex (ref: boy)		Migration background (ref: no)		Maternal education (ref: high)	
Intervention = Outdoor play	B	95% CI	B	95% CI	B	95% CI
Before intervention	0.106	(0.065, 0.145)	0.125	(0.085, 0.165)	0.177	(0.138, 0.216)
After intervention	0.118	(0.050, 0.188)	0.130	(0.063, 0.199)	0.184	(0.117, 0.252)
Change	0.013	(−0.045, 0.071)	0.005	(−0.051, 0.063)	0.007	(−0.050, 0.064)
Intervention = Sports participation						
Before intervention	0.106	(0.066, 0.145)	0.125	(0.085, 0.165)	0.177	(0.138, 0.216)
After intervention	0.097	(0.058, 0.136)	0.120	(0.081, 0.160)	0.163	(0.125, 0.202)
Change	−0.009	(−0.020, 0.002)	−0.005	(−0.017, 0.006)	**−0.014**	**(−0.024**,**−0.003)**
Intervention = Screen time						
Before intervention	0.106	(0.066, 0.145)	0.125	(0.085, 0.165)	0.177	(0.139, 0.216)
After intervention	0.094	(0.052, 0.137)	0.126	(0.083, 0.169)	0.160	(0.117, 0.202)
Change	−0.011	(−0.032, 0.010)	0.001	(−0.021, 0.023)	−0.017	(−0.039, 0.004)

Externalizing symptoms						
	Sex (ref: boy)		Migration background (ref: no)		Maternal education (ref: high)	
Intervention = Outhoor play	B	95% CI	B	95% CI lower bound	B	95% CI
Before intervention	−0.154	(−0.193, −0.114)	0.097	(0.057, 0.137)	0.154	(0.116, 0.193)
After intervention	−0.153	(−0.220, −0.085)	0.076	(0.007, 0.146)	0.165	(0.097, 0.235)
Change	0.000	(−0.052, 0.054)	−0.021	(−0.075, 0.034)	0.011	(−0.043, 0.066)
Intervention = Sports participation						
Before intervention	−0.154	(−0.193, −0.114)	0.097	(0.057, 0.137)	0.154	(0.115, 0.193)
After intervention	−0.157	(−0.197, −0.118)	0.096	(0.056, 0.137)	0.149	(0.110, 0.189)
Change	−0.004	(−0.013, 0.006)	0.000	(−0.011, 0.010)	−0.005	(−0.015, 0.005)
Intervention = Screen time						
Before intervention	−0.154	(−0.193, −0.114)	0.097	(0.057, 0.137)	0.154	(0.115, 0.193)
After intervention	−0.169	(−0.211, −0.128)	0.095	(0.053, 0.138)	0.140	(0.098, 0.181)
Change	−0.016	(−0.035, 0.004)	−0.002	(−0.023, 0.019)	−0.015	(−0.034, 0.005)

Significance means that zero does not fall within the Confidence Interval

*Ref* reference group, *B* standardized beta coefficient, *CI* Confidence Interval

### Externalizing symptoms

Boys had a 0.154 (95% CI: 0.114, 0.193) standard deviation more externalizing symptoms compared to girls. Similarly, children with a migration background had a 0.097 (95% CI: 0.057, 0.137) standard deviation more externalizing symptoms than those without, and children with a low-educated mother had a 0.154 (95% CI: 0.115; 0.193) standard deviation more externalizing symptoms.

In the main analyses, none of the hypothetical interventions significantly reduced any of the social disparities (Table 2). In the sensitivity analyses, hypothetically increasing sports participation and decreasing screen time reduced the disparities for maternal education in the complete case sample (β = -0.020; 95% CI: -0.036, -0.004; and β = -0.034; 95% CI: -0.072, 0.000 respectively; Table S4), and the self-reported externalizing symptoms (β = 0.018; 95% CI: 0.006, 0.030; and β = 0.025; 95% CI: 0.003, 0.048 respectively; Table S5). Interestingly, hypothetically reducing screen time increased disparities in externalizing symptoms between the sexes in the complete case sample (β = 0.041; 95% CI: 0.014, 0.071) (Table S4), and hypothetically increasing sports participation also increased the disparities in self-reported externalizing symptoms between the sexes (Table S5).

## Discussion

Using a sequential G-estimation model, this study aimed to estimate how social disparities in child mental health at age 13 might change following hypothetical interventions targeting physical activity and screen time at age 10. The study found that children with a migration background and children with a lower-educated mother reported more internalizing and externalizing symptoms than children without a migration background and children with a higher-educated mother. In contrast, girls reported more internalizing symptoms than boys, while boys experienced more externalizing symptoms than girls. Hypothetically increasing outdoor play and reducing screen time did not lead to a statistically significant reduction in disparities in internalizing symptoms. However, hypothetically increasing sports participation led to a statistically significant reduction in disparities in internalizing symptoms related to maternal education. The study also indicated that increasing sports participation or reducing screen time has varying impacts on social disparities in externalizing symptoms, with certain interventions reducing maternal educational disparities, but increasing sex disparities in some of the sensitivity analyses, denoting inconsistent results when discussing externalizing symptoms.

A hypothetical increase in sports participation lead to a statistically significant attenuation of disparities in internalizing symptoms associated with maternal education. This finding aligns with our previous research, which demonstrated that sports participation was inversely associated with internalizing symptoms in youth, although the observed effects were modest [27]. Consistent with other earlier studies, the magnitude of the associations in our research corresponds with findings showing that involvement in sports during childhood is linked to fewer depressive symptoms in early adulthood, although again the effect sizes were small after accounting for confounders [10, 28, 29]. Evidence also suggests that the beneficial impact of physical activity on psychiatric symptoms is more pronounced in controlled clinical samples compared to general population studies [28]. Despite this, physical activity consistently emerges as a valuable tool contributing to preventing youth from developing clinical or diagnosed mental disorders, particularly in the critical developmental period of adolescence.

Home environments characterized by low parental education or low socioeconomic status often act as early life adversities, contributing to the emergence of psychiatric problems in childhood. However, some children in similar circumstances may exhibit greater resistance to the development of psychiatric symptoms, which may be partially explained by intrapersonal factors such as IQ, self-identity, and self-esteem [30–32]. Research suggests that self-esteem can act as a protective factor against the development of psychiatric symptoms in the face of early adversities, and sport-based interventions have been shown to improve self-esteem [33]. This improvement could play a role in mitigating the risk of internalizing symptoms among youth exposed to early life adversities [30].

Not all forms of physical activity appear to offer the same benefits. Outdoor play may have a smaller impact than sports, particularly sports focused on skill development and teamwork rather than aesthetics [27, 34]. Previous evidence indicates that structured sports, particularly team-based activities, foster self-esteem and social connectedness, key protective factors against internalizing symptoms [34]. These findings align with the notion that the quality and structure of physical activity, rather than its sheer quantity, are central to its mental health benefits, which may explain the contrasting findings observed between organized sports and unstructured outdoor play.

No statistically significant changes in social or sex disparities in psychiatric symptoms were observed following a hypothetical intervention aimed at reducing screen time to two hours per day. Overall, boys spent more time using screens than girls. Marked differences were also found between children without and with a migration background, as well as between children of mothers with low and high educational attainment. The hypothetical intervention reducing screen time to two hours per day led to a substantially larger reduction among boys, children with a migration background, and children of less educated mothers compared with their respective reference groups. Despite these differences in the magnitude of reduction, no statistically significant changes in psychiatric symptoms were detected. We hypothesize that this null finding may be due to limitations in the screen time measures used at the time of data collection. Specifically, the measure included only television viewing and video gaming, excluding other, now more prevalent, screen-based activities such as social media use (e.g., Instagram, TikTok), which represent a major portion of screen engagement among youth [35, 36]. Additionally, the content and context of screen use were not assessed. Prior research has shown that the type of on-screen content may play a crucial role in its psychological impact, with exposure to educational or age-appropriate material being associated with more favorable outcomes compared to non-educational or violent content [37, 38]. However, other studies have reported that these differences account for only small variations in mental health indicators, suggesting that the influence of screen content may be limited [39, 40]. Furthermore, many other studies in this field face similar methodological limitations—such as relying on self-reported data, focusing on only certain types of screen use, or neglecting contextual and content-related factors—which may help explain the overall weak and inconsistent evidence regarding the effects of screen time on psychiatric symptoms [41]. Another possible explanation is that there may simply be little or no causal association between total screen time and mental health outcomes. Recent meta-analyses and longitudinal studies have reported that associations between screen exposure and psychiatric symptoms tend to be small and often attenuate after accounting for confounding variables such as pre-existing mental health, socioeconomic background, or genetic factors [42, 43]. Therefore, while reducing screen time may have other benefits, the direct impact on psychiatric symptoms remains uncertain, underscoring the need for future studies to employ more comprehensive and context-sensitive measures of screen engagement.

The impact of these hypothetical interventions on externalizing symptoms was inconsistent, with non-statistically significant results in the main analyses. However, sensitivity analyses using complete cases and self-reported data revealed that some interventions reduced disparities among children with lower maternal education, while others led to an increase in sex disparities. The inconsistencies observed in the impact of hypothetical interventions on social disparities in externalizing symptoms may be influenced by several factors. First, the relationship between physical activity variables and externalizing symptoms may be non-statistically significant or weak, which could explain some of the negative results observed in our main analyses [27]. Additionally, the CBCL, a widely used and validated instrument to measure psychopathology in children, may have limitations that affect the accuracy of measuring externalizing symptoms. Specifically, although the majority (87.4%) of CBCL items are not biased by gender, some items related to externalizing symptoms, particularly those assessing aggressive behavior, have been identified as potentially gender-biased in previous studies [7]. Another potential explanation for the discrepancies found could be the use of sex rather than gender as an inequality measure in our study, as it may not account for the continuous nature of gender identity, potentially affecting our results. Overall, these limitations could help explain our inconsistent findings regarding differences between boys and girls and contribute to some of the discrepancies observed in the results. Furthermore, it is possible that the CBCL measures of externalizing symptoms in youth may not be sensitive enough to detect subtle or nuanced differences in behavior. All in all, addressing these measurement issues could lead to a better understanding of the relationship between physical activity and externalizing symptoms. However, our findings might also be accurate: lifestyle interventions could reduce SES-related disparities in externalizing behavior while unintentionally exacerbating sex disparities. This outcome may reflect differences in how boys and girls respond to interventions due to biological, social, or environmental factors. Careful evaluation and tailoring of interventions are crucial to addressing both SES and sex disparities, ensuring equitable outcomes across groups.

This study is among the first to employ this methodology to quantify reductions in social disparities in child mental health at age 10 through hypothetical interventions targeting outdoor play, sports participation, and screen time. Recent advancements in causal inference methodology have substantially improved the study of health disparities, offering tools to directly assess the key parameter of interest: the potential for interventions to reduce these disparities [19, 44]. Unlike many studies that adjust for confounders in the relationship between social disparities and health outcomes, this study intentionally avoids such adjustments. The rationale is that unadjusted health disparities are of primary interest, as they represent real-world disparities in health outcomes based on factors like sex, maternal education, or migration background, rather than disparities conditioned on covariates. By focusing on unadjusted health disparities, this approach provides a clearer understanding of how specific interventions could influence social disparities and sets realistic expectations for their effect sizes. Additional strengths of this study include the use of a population-based, multicultural cohort, which enhances the generalizability of the findings, and the application of multiple imputation techniques to minimize selection bias and ensure representative data. These methodological strengths contribute to a more comprehensive understanding of strategies to address social disparities in child mental health.

Nonetheless, our findings must be interpreted in the context of several limitations. First, the observational design restricts our ability to infer causality for any of the hypothetical interventions and their outcomes. Second, other potential confounders not included in the model may also contribute to reducing the social disparities explored in this study. Third, we measured both the hypothetical interventions and the outcomes at a single time point, which prevented us from examining the stability of these variables from childhood to adolescence. Fourth, hypothetical interventions were assessed through parental reports, which introduces the possibility of under- or overestimations of behaviors. Furthermore, both the hypothetical interventions and the outcomes were reported by the primary caregivers, which may have inflated the observed relationships due to shared method variance. Sensitivity analyses indicated that when the child reported on the outcomes, some results, particularly those related to externalizing symptoms, showed slight changes, while those related to internalizing symptoms remained consistent. Fifth, this study assesses sex (assigned at birth) rather than gender, which may limit the ability to capture the potential association of gender identity and expression with psychiatric symptoms. Sixth, despite the study´s multi-ethnic and diverse composition which represents the city of Rotterdam and other larger cities in the Netherlands, dropout was more common among participants with lower maternal education and with migration background (Table 1). In order to deal with attrition bias, we used the whole cohort (*N* = 9,898) in the main analyses and imputed the missing values using multivariate imputation by chained equations [24]. Lastly, while we used multiple imputations to ensure all participants were included in the analysis and reduce bias, the results may still be influenced by missing data, especially if the missingness is related to variables not included in the imputation model. We sought to mitigate this by incorporating all variables from the analyses, as well as additional variables associated with missingness, into the imputation process.

### Practical implications and future research

Given that many internalizing problems, including depression, anxiety, and somatic symptoms, typically emerge during formative years—especially among young people from low-education and migrant backgrounds, as well as those affected by sex differences —investing in early interventions targeting at-risk youth is essential [2]. New societal challenges, such as the COVID-19 pandemic, climate change, and the ongoing war in Ukraine, may exacerbate the risk of mental health issues [45]. In light of these growing concerns, policymakers should prioritize programs that encourage physical activity, particularly sports, as part of a broader strategy to mitigate the escalating mental health burden worldwide. This approach is especially important for youth from lower socioeconomic backgrounds to ensure they have equal opportunities to thrive compared to their peers with higher socioeconomic status. Future intervention studies focusing on these at-risk populations are essential to determine the most cost-effective delivery methods—whether through online, blended, or in-person approaches. These interventions should aim not only to provide young people with access to sports but also to integrate behavioral change strategies that promote a sustained positive impact on mental health.

## Conclusion

In conclusion, this study highlights the presence of social disparities in both internalizing and externalizing symptoms in young people, with sex, migration background, and maternal education playing a key role. While hypothetical interventions targeting outdoor play and screen time did not uniformly reduce these disparities, increasing sports participation seems consistently effective in reducing disparities in internalizing symptoms related to maternal education. The impact of hypothetical lifestyle interventions on externalizing symptoms was inconsistent, with some interventions showing reductions in disparities for maternal education but increasing sex disparities. These findings suggest that while certain lifestyle interventions can reduce disparities in child mental health, the effects may vary across different types of symptoms and demographic groups, emphasizing the need for tailored approaches in addressing social disparities in mental health. Future research should further explore the differential effects of interventions on internalizing and externalizing symptoms across various social contexts.

## Supplementary Material

Below is the link to the electronic supplementary material.

Supplementary Material 1

## Data Availability

Data from this study are available upon reasonable request to the director of the Generation R Study (generationr@erasmusmc.nl), subject to local, national and European rules and regulations.
