# Supplementary material for: Reducing social disparities in child emotional and behavioral problems by hypothetical physical activity and screen time interventions
Source: Soc Psychiatry Psychiatr Epidemiol. 2026 Jan 19;61(5):819–28. doi: 10.1007/s00127-025-03036-6 (PMC13156153; doi:10.1007/s00127-025-03036-6)
Supplement: Supplementary file 1 — Supplementary Material 1 [file 127_2025_3036_MOESM1_ESM.docx]

**Supplementary Material**

**Table S1.** Characteristics of the cohort stratified by sex.

|  | **Boys (N=4,937)** | **Girls (N=4,808)** | **(Missing) (N=153)** |
| --- | --- | --- | --- |
| **Migration background** |  |  |  |
| No | 2,488 (53.9%) | 2,406 (53.8%) | 54 (41.2%) |
| Yes | 2,131 (46.1%) | 2,065 (46.2%) | 77 (58.8%) |
| N | 4,619 | 4,471 | 131 |
| **Maternal education** |  |  |  |
| Lower | 2,425 (53.1%) | 2,355 (53.2%) | 78 (62.4%) |
| Higher | 2,144 (46.9%) | 2,073 (46.8%) | 47 (37.6%) |
| N | 4,569 | 4,428 | 125 |
| **Child age at psychiatric measurement** |  |  |  |
| Mean (SD) | 13.56 (0.40) | 13.56 (0.40) | NA |
| N | 2,454 | 2,507 | 0 |
| **Outdoor play (hrs/week)** |  |  |  |
| Mean (SD) | 6.09 (4.78) | 5.18 (4.48) | NA |
| N | 2,401 | 2,410 | 0 |
| **Sports participation (hrs/week)** |  |  |  |
| Mean (SD) | 2.84 (1.59) | 2.36 (1.54) | NA |
| N | 2,444 | 2,461 | 0 |
| **Screen time (hrs/week)** |  |  |  |
| Mean (SD) | 18.48 (12.54) | 16.09 (11.11) | NA |
| N | 2,212 | 2,212 | 0 |
| **Internalizing symptoms (sumscore)** |  |  |  |
| Mean (SD) | 5.26 (5.47) | 5.99 (6.11) | NA |
| N | 2,343 | 2,378 | 0 |
| **Externalizing symptoms (sumscore)** |  |  |  |
| Mean (SD) | 4.65 (5.61) | 3.80 (4.90) | NA |
| N | 2,335 | 2,374 | 0 |
| **Self-reported internalizing symptoms (sumscore)** |  |  |  |
| Mean (SD) | 7.27 (6.10) | 10.21 (7.75) | NA |
| N | 2,203 | 2,313 | 0 |
| **Self-reported externalizing symptoms (sumscore)** |  |  |  |
| Mean (SD) | 7.53 (5.46) | 6.57 (5.16) | NA |
| N | 2,191 | 2,312 | 0 |

Hrs= Hours. N= Number of participants. SD= Standard deviation

**Table S2.** Characteristics of the cohort stratified by migration background.

|  | **No migration background (N=4,937)** | **Migration background (N=4,808)** | **(Missing) (N=153)** |
| --- | --- | --- | --- |
| **Sex** |  |  |  |
| Boy | 2,488 (50.8%) | 2,131 (50.8%) | 318 (48.5%) |
| Girl | 2,406 (49.2%) | 2,065 (49.2%) | 337 (51.5%) |
| N | 4,894 | 4,196 | 655 |
| **Maternal education** |  |  |  |
| Lower | 1,998 (40.5%) | 2,831 (68.2%) | 29 (72.5%) |
| Higher | 2,935 (59.5%) | 1,318 (31.8%) | 11 (27.5%) |
| N | 4,933 | 4,149 | 40 |
| **Child age at psychiatric measurement** |  |  |  |
| Mean (SD) | 13.53 (0.37) | 13.61 (0.45) | 13.64 (0.37) |
| N | 3213 | 1698 | 50 |
| **Outdoor play (hrs/week)** |  |  |  |
| Mean (SD) | 5.78 (4.54) | 5.34 (4.84) | 6.30 (5.11) |
| N | 3111 | 1649 | 51 |
| **Sports participation (hrs/week)** |  |  |  |
| Mean (SD) | 2.82 (1.50) | 2.19 (1.66) | 2.60 (1.65) |
| N | 3163 | 1691 | 51 |
| **Screen time (hrs/week)** |  |  |  |
| Mean (SD) | 15.43 (9.53) | 20.96 (14.94) | 20.81 (14.33) |
| N | 2938 | 1441 | 45 |
| **Internalizing symptoms (sumscore)** |  |  |  |
| Mean (SD) | 5.37 (5.65) | 6.06 (6.02) | 7.86 (7.60) |
| N | 3076 | 1599 | 46 |
| **Externalizing symptoms (sumscore)** |  |  |  |
| Mean (SD) | 4.04 (5.02) | 4.56 (5.69) | 5.17 (6.53) |
| N | 3069 | 1594 | 46 |
| **Self-reported internalizing symptoms (sumscore)** |  |  |  |
| Mean (SD) | 8.67 (7.02) | 8.94 (7.36) | 10.35 (8.32) |
| N | 2964 | 1511 | 41 |
| **Self-reported externalizing symptoms (sumscore)** |  |  |  |
| Mean (SD) | 7.02 (5.22) | 7.07 (5.54) | 6.57 (5.53) |
| N | 2959 | 1503 | 41 |

Hrs= Hours. N= Number of participants. SD= Standard deviation

**Table S3.** Characteristics of the cohort stratified by maternal education.

|  | **Lower maternal education (N=4,858)** | **Higher maternal education (N=4,264)** | **(Missing) (N=776)** |
| --- | --- | --- | --- |
| **Sex** |  |  |  |
| Boy | 2,425 (50.7%) | 2,144 (50.8%) | 368 (49.2%) |
| Girl | 2,355 (49.3%) | 2,073 (49.2%) | 380 (50.8%) |
| N | 4,780 | 4,217 | 748 |
| **Migration background** |  |  |  |
| No | 1,998 (41.4%) | 2,935 (69.0%) | 15 (10.8%) |
| Yes | 2,831 (58.6%) | 1,318 (31.0%) | 124 (89.2%) |
| N | 4,829 | 4,253 | 139 |
| **Child age at psychiatric measurement** |  |  |  |
| Mean (SD) | 13.58 (0.44) | 13.54 (0.37) | 13.72 (0.58) |
| N | 1,940 | 2,957 | 64 |
| **Outdoor play (hrs/week)** |  |  |  |
| Mean (SD) | 6.14 (5.07) | 5.30 (4.33) | 5.63 (4.76) |
| N | 1,885 | 2,864 | 62 |
| **Sports participation (hrs/week)** |  |  |  |
| Mean (SD) | 2.17 (1.62) | 2.88 (1.49) | 2.48 (1.65) |
| N | 1,930 | 2,911 | 64 |
| **Screen time (hrs/week)** |  |  |  |
| Mean (SD) | 21.27 (14.43) | 14.66 (9.02) | 21.35 (13.04) |
| N | 1,704 | 2,666 | 54 |
| **Internalizing symptoms (sumscore)** |  |  |  |
| Mean (SD) | 6.10 (6.07) | 5.27 (5.57) | 8.41 (7.52) |
| N | 1,823 | 2,841 | 57 |
| **Externalizing symptoms (sumscore)** |  |  |  |
| Mean (SD) | 4.59 (5.64) | 3.96 (4.99) | 5.64 (6.74) |
| N | 1,818 | 2,836 | 55 |
| **Self-reported internalizing symptoms (sumscore)** |  |  |  |
| Mean (SD) | 9.05 (7.37) | 8.56 (6.95) | 10.95 (8.83) |
| N | 1,737 | 2,726 | 53 |
| **Self-reported externalizing symptoms (sumscore)** |  |  |  |
| Mean (SD) | 7.18 (5.85) | 6.95 (4.97) | 6.77 (5.53) |
| N | 1,724 | 2,726 | 53 |

Hrs= Hours. N= Number of participants. SD= Standard deviation.

| **Table S4.** Reductions in sex, migration background and maternal education disparities on internalizing and externalizing symptoms at the age of 13 from hypothetical outdoor play, sports participation and screen time interventions in children at age 10. Analysis with complete cases (N=3,413). | | | | | | | | | | |
| --- | --- | --- | --- | --- | --- | --- | --- | --- | --- | --- |
| **Internalizing symptoms** | | | | | | | | | | |
|  | **Sex (ref:boy)** | | | **Migration background (ref:no)** | | | **Maternal education (ref:high)** | | | |
| Intervention= Outdoor play | **B** | **95% CI** | | **B** | **95% CI** | | **B** | | **95% CI** | |
| Before intervention | 0.087 | (0.027, | 0.148) | 0.094 | (0.027, | 0.162) | 0.091 | | (0.028, | 0.155) |
| After intervention | 0.172 | (0.057, | 0.289) | 0.088 | (-0.034, | 0.213) | 0.110 | | (-0.005, | 0.229) |
| Change | 0.085 | (-0.016, | 0.186) | -0.006 | (-0.111, | 0.099) | 0.019 | | (-0.081, | 0.122) |
| Intervention= Sports participation |  |  |  |  |  |  |  | |  |  |
| Before intervention | 0.087 | (0.028, | 0.148) | 0.094 | (0.026, | 0.162) | 0.091 | | (0.027, | 0.156) |
| After intervention | 0.080 | (0.022, | 0.140) | 0.091 | (0.023, | 0.157) | 0.074 | | (0.012, | 0.138) |
| Change | -0.007 | (-0.021, | 0.006) | -0.003 | (-0.023, | 0.016) | **-0.017** | | **(-0.034,** | **-0.000)** |
| Intervention= Screen time |  |  |  |  |  |  |  | |  |  |
| Before intervention | 0.087 | (0.027, | 0.148) | 0.094 | (0.026, | 0.162) | 0.091 | | (0.027, | 0.155) |
| After intervention | 0.067 | (0.004, | 0.130) | 0.110 | (0.036, | 0.185) | 0.067 | | (-0.002, | 0.136) |
| Change | -0.020 | (-0.049, | 0.008) | 0.017 | (-0.021, | 0.054) | -0.024 | | (-0.060, | 0.012) |
| **Externalizing symptoms** | | | | | | | | | | |
|  | **Sex (ref:boy)** | | | **Migration background (ref:no)** | | | **Maternal education (ref:high)** | | | |
| Intervention= Outdoor play | **B** | **95% CI** | | **B** | **95% CI** | | **B** | **95% CI** | | |
| Before intervention | -0.160 | (-0.218, | -0.101) | 0.094 | (0.027, | 0.163) | 0.079 | (0.017, | | 0.142) |
| After intervention | -0.119 | (-0.235, | -0.003) | 0.056 | (-0.074, | 0.194) | 0.081 | (-0.038, | | 0.201) |
| Change | 0.040 | (-0.053, | 0.135) | -0.038 | (-0.142, | 0.071) | 0.003 | (-0.093, | | 0.100) |
| Intervention= Sports participation |  |  |  |  |  |  |  |  | |  |
| Before intervention | -0.160 | (-0.218, | -0.101) | 0.094 | (0.026, | 0.164) | 0.079 | (0.016, | | 0.141) |
| After intervention | -0.170 | (-0.227, | -0.112) | 0.090 | (0.021, | 0.161) | 0.059 | (-0.004, | | 0.121) |
| Change | -0.010 | (-0.022, | 0.001) | -0.004 | (-0.022, | 0.013) | **-0.020** | **(-0.036,** | | **-0.004)** |
| Intervention= Screen time |  |  |  |  |  |  |  |  | |  |
| Before intervention | -0.160 | (-0.218, | -0.101) | 0.094 | (0.027, | 0.165) | 0.079 | (0.017, | | 0.141) |
| After intervention | -0.201 | (-0.263, | -0.139) | 0.093 | (0.019, | 0.169) | 0.044 | (-0.021 | | 0.112,) |
| Change | **-0.041** | **(-0.071,** | **-0.014)** | -0.001 | (-0.038, | 0.034) | **-0.034** | **(-0.072,** | | **-0.000)** |

Ref= reference group. B= standardized beta coefficient. CI= Confidence Interval.

| **Table S5.** Reductions in sex, migration background and maternal education disparities on self-reported internalizing and externalizing symptoms at the age of 13 from hypothetical outdoor play, sports participation and screen time interventions in children at age 10. | | | | | | | | | |
| --- | --- | --- | --- | --- | --- | --- | --- | --- | --- |
| **Internalizing symptoms** | | | | | | | | | |
|  | **Sex (ref:boy)** | | | **Migration background (ref:no)** | | | **Maternal education (ref:high)** | | |
| **Intervention= Outdoor play** | **B** | **95% CI** | | **B** | **95% CI** | | **B** | **95% CI** | |
| Before intervention | 0.380 | (0.341, | 0.420) | 0.051 | (0.011, | 0.091) | 0.105 | (0.065, | 0.145) |
| After intervention | 0.360 | (0.282, | 0.438) | 0.062 | (-0.012, | 0.136) | 0.127 | (0.054, | 0.201) |
| Change | -0.021 | (-0.090, | 0.048) | 0.011 | (-0.054, | 0.075) | 0.022 | (-0.042, | 0.087) |
| **Intervention= Sports participation** |  |  |  |  |  |  |  |  |  |
| Before intervention | 0.380 | (0.342, | 0.419) | 0.051 | (0.011, | 0.091) | 0.105 | (0.066, | 0.145) |
| After intervention | 0.364 | (0.326, | 0.403) | 0.039 | (-0.001, | 0.079) | 0.081 | (0.042, | 0.121) |
| Change | **-0.016** | **(-0.029,** | **-0.003)** | **-0.012** | **(-0.025,** | **0.000)** | **-0.024** | **(-0.036,** | **-0.012)** |
| **Intervention= Screen time** |  |  |  |  |  |  |  |  |  |
| Before intervention | 0.380 | (0.341, | 0.420) | 0.051 | (0.011, | 0.091) | 0.105 | (0.065, | 0.144) |
| After intervention | 0.368 | (0.326, | 0.411) | 0.035 | (-0.009, | 0.079) | 0.075 | (0.031, | 0.117) |
| Change | -0.012 | (-0.036, | 0.011) | -0.017 | (-0.040, | 0.007) | **-0.030** | **(-0.052,** | **-0.009)** |
| **Externalizing symptoms** | | | | | | | | | |
|  | **Sex (ref:boy)** | | | **Migration background (ref:no)** | | | **Maternal education (ref:high)** | | |
| **Intervention= Outdoor play** | **B** | **95% CI** | | **B** | **95% CI** | | **B** | **95% CI** | |
| Before intervention | -0.186 | (-0.226, | -0.147) | 0.026 | (-0.014, | 0.066) | 0.092 | (0.053, | 0.131) |
| After intervention | -0.225 | (-0.306, | -0.145) | 0.022 | (-0.060, | 0.104) | 0.118 | (0.038, | 0.198) |
| Change | -0.039 | (-0.106, | 0.028) | -0.004 | (-0.071, | 0.063) | 0.027 | (-0.041, | 0.094) |
| **Intervention= Sports participation** |  |  |  |  |  |  |  |  |  |
| Before intervention | -0.186 | (-0.226, | -0.147) | 0.026 | (-0.014, | 0.066) | 0.099 | (0.053, | 0.131) |
| After intervention | -0.198 | (-0.238, | -0.158) | 0.021 | (-0.019, | 0.063) | 0.074 | (0.034, | 0.114) |
| Change | **-0.012** | **(-0.024,** | **-0.000)** | -0.005 | (-0.017, | 0.008) | **-0.018** | **(-0.030,** | **-0.006)** |
| **Intervention= Screen time** |  |  |  |  |  |  |  |  |  |
| Before intervention | -0.186 | (-0.225, | -0.147) | 0.026 | (-0.014, | 0.066) | 0.091 | (0.053, | 0.131) |
| After intervention | -0.203 | (-0.245, | -0.159) | 0.019 | (-0.025, | 0.064) | 0.066 | (0.023, | 0.110) |
| Change | -0.017 | (-0.040, | 0.006) | -0.007 | (-0.031, | 0.017) | **-0.025** | **(-0.048,** | **-0.003)** |

Ref= reference group. B= standardized beta coefficient. CI= Confidence Interval.
